# Indoor Passive Visual Positioning by CNN-Based Pedestrian Detection

**DOI:** 10.3390/mi13091413

**Published:** 2022-08-27

**Authors:** Dewen Wu, Ruizhi Chen, Yue Yu, Xingyu Zheng, Yan Xu, Zuoya Liu

**Affiliations:** 1 State Key Laboratory of Information Engineering in Surveying, Mapping and Remote Sensing (LIESMARS), Wuhan University, Wuhan 430079, China; 2 Collaborative Innovation Center of Geospatial Technology, Wuhan University, Wuhan 430079, China

**Keywords:** passive visual positioning, pedestrian detection, region of interest attention model, projection transformation

## Abstract

Indoor positioning applications are developing at a rapid pace; active visual positioning is one method that is applicable to mobile platforms. Other methods include Wi-Fi, CSI, and PDR approaches; however, their positioning accuracy usually cannot achieve the positioning performance of the active visual method. Active visual users, however, must take a photo to obtain location information, raising confidentiality and privacy issues. To address these concerns, we propose a solution for passive visual positioning based on pedestrian detection and projection transformation. This method consists of three steps: pretreatment, pedestrian detection, and pose estimation. Pretreatment includes camera calibration and camera installation. In pedestrian detection, features are extracted by deep convolutional neural networks using neighboring frame detection results and the map information as the region of interest attention model (RIAM). Pose estimation computes accurate localization results through projection transformation (PT). This system relies on security cameras installed in non-private areas so that pedestrians do not have to take photos. Experiments were conducted in a hall about 100 square meters in size, with 41 test-points for the localization experiment. The results show that the positioning error was 0.48 m (RMSE) and the 90% error was 0.73 m. Therefore, the proposed passive visual method delivers high positioning performance.

## 1. Introduction

With the application and development of technologies based on user positioning information, location-based services are now growing at a rapid pace. However, after decades of research, there are still no reliable products for indoor localization, although demand is rapidly increasing [1]. Most of the work on indoor localization focuses on the provision of a widely applicable scheme for indoor localization such as GPS. The global navigation satellite system (GNSS) can provide excellent location accuracy and can be fully used in outdoor environments, but in indoor environments, its signals are weak and easily blocked or attenuated by buildings [2]. Therefore, indoor localization is still a challenging and meaningful research topic [3].

Generally, indoor positioning methods fall into two categories. Some are focused on fingerprinting-based localization algorithms, given their effectiveness and independence of infrastructure [4,5,6]. Fingerprinting-based methods include Wi-Fi and magnetic fingerprinting. Wi-Fi received signal strengths (RSS) or magnetic field strengths (MFS) are collected and compared with data in a fingerprinting database based on the assumption that each location has a unique signal feature [7]. This kind of scheme is easy to establish and can achieve fine localization performance, but the signal patterns change over time and are vulnerable to environmental changes, which means this method is only reliable for a short term [8]. Furthermore, RSS or MFS require labor-intensive and time-consuming data collection, and there are many situations where different locations have the same signal patterns or a similar signal feature. To overcome the defects of these schemes, other schemes have been proposed, such as radio frequency identification (RFID), pseudo-satellite, optical, 5G, etc. However, the accuracy and robustness of these systems are not enough for wide use due to the complexities of indoor environments.

For example, simultaneous localization and mapping (SLAM) relies on a variety of sensors in practical applications. Real-time location calculation and reconstruction require high computing power and expensive equipment [9,10]. Moreover, positioning based on image library matching requires matching photos taken by pedestrians and images in the image library. This method needs to build different image databases for different scenes [11]. Pedestrians must take photos, raising privacy issues and inevitably leading to differences in image quality, which reduce positioning accuracy.

To overcome the limitations of active visual localization, we proposed a passive vision scheme. The passive visual positioning method exploits security cameras mounted in public areas. Only police officers and administrators can see the pictures taken by security cameras. Users just obtain their position and do not access the pictures, so this method avoids confidentiality and privacy problems. In this study, we demonstrate passive visual localization based on pedestrian detection and projection transformation, which does not require users to take a photo and can achieve sub-meter level localization accuracy. In this system, security cameras set at a certain height capture pictures of the monitoring area in real time. A server obtains the real-time pictures from the Wi-Fi and performs a computing operation to extract accurate and precise pedestrian location information.

The main contributions of this study can be summarized as follows:

1. Our visual localization algorithm is a passive vision scheme. Unlike active visual localization schemes, users do not need to take photos when they want to obtain their position.
2. Our visual localization algorithm makes full use of inter-frame detection results and CAD map information. Unlike other visual detection methods, we propose a new spatial attention model that fuses CAD map information and detection results from previous frames. We call this model the region of interest attention model (RIAM).
3. Our visual localization algorithm is not limited to the detection results. Compared with other visual detection methods, not only do we know if there are people in the scene, but we also know exactly where the pedestrians are. We recover individual 2D positions from the pedestrian detection pixel results.
4. Our detection method achieved 83.49% detection accuracy, representing an increase of about 1.56% over the YOLOv3 approach. Compared with the YOLOv3 +PT method, our passive visual positioning method increased average accuracy by about 12.2%, achieved an average accuracy of 40.4 cm, and about 90% of the results were less than 73.2 cm from the actual position.

The paper proceeds as follows: Section 2 provides a brief overview of related work. The system overview and methods are described in detail in Section 3. Experiment evaluations are presented in Section 4. Section 5 is the conclusion, where we discuss some potential future research.

## 2. Related Work

Our task of indoor localization is related to several areas of recent interest in computer vision, such as visual localization, target detection, and visual pose estimation.

At present, visual localization systems can be roughly divided into two categories. Active visual positioning methods are the most common visual localization methods, whereby users must use photographic imaging equipment to build a relationship between themselves and surrounding environments. However, passive visual localization methods are based on security cameras so that users do not have to take photos, as the security camera can build the relationship between pedestrians and the surrounding environments. Furthermore, active visual positioning systems are divided into two types: matching-based localization methods and image-database-based localization [12].

Matching-based localization methods are the most common visual localization methods that use point, line, or optical flows to estimate image-to-image matches between a picture and the image database or between a picture and the continuous image. Camera localization can be estimated by a collinear equation and the correspondence of several images. In 2004, Lowe proposed the scale-invariant feature transform (SIFT), which can resist the influence caused by rotation, translation, scaling, viewpoint, illumination, and so on [13]. However, the SIFT incurs large time costs. Rublee proposed the oriented fast and rotated BRIEF (ORB) in 2011 [14]. Although this method may cause much mismatching, it is about 100 times faster than SIFT. Line-matching methods are used in environments without enough texture features-. In 2013, Zhang proposed the line band descriptor (LBD), which is similar to the mean–standard deviation line descriptor (MSLD) method. LBD outperforms MSLD in illumination, rotation, occlusion, and low-texture scenarios, but is more sensitive to scale [15,16,17]. An optical flow method for matching images was proposed in 1950 by Gibson. Since then, there have been many improvements such as the Lucas–Kanade (LK) optical flow proposed in 1999 and the optical flow proposed in 2015 [18,19]. Structure from motion (SfM) can estimate camera position based on matching. Torsten proposed a combination of 2D-based methods with local SfM reconstruction, which has both a simple database construction procedure and yields accurate pose estimation [20]. Zhang [21] fused the data from an RGB-D camera and an inertial measurement unit (IMU).

Localization methods based on image databases rely on repositories of public geo-labeled images [22]. This method compares the photo with the geo-labeled images to choose the closest match, and the localization of the photo is estimated by the most similar image from the geo-labeled images database [23,24,25,26]. The quality of geo-labeled images is the core of these methods, including shape, color, and texture [27]. Zhang [28] proposed the improved vector of local aggregated descriptors to improve the retrieval accuracy under different lighting conditions. Most of the conventional methods retrieve images based on local descriptor matching and reorder them through elaborate spatial verification [29,30,31]. Pretrained deep convolution neural networks may effectively extract features [32,33,34,35]. Yu [36] used the SSD convolution neural networks to extract the features to match. Nevertheless, the major drawback of active visual positioning systems is the requirement that users must take a photo, which may raise privacy issues; in addition, the quality of a photograph is not stable, which will affect localization accuracy.

Passive visual localization methods mostly consist of detection-based localization solutions. Detection-based localization methods are based on the ubiquitous security cameras surrounding us in everyday life. It is quite different from other visual localization methods as users are being photographed, so target detection is necessary. Target detection is a visual task that detects and labels the position of objects from an image [37]. Conventional methods detect objects based on features; these include the Viola–Jones detector (VJ), directional gradient histogram detector (HOG), deformable-part-based model (DPM), and so on [38,39,40,41]. Recent works leverage deep convolution neural networks for target detection; they are all becoming faster and more accurate [42]. Ross proposed regions with CNN (R-CNN) in 2014; this method determines in advance where the target might appear, thus avoiding a naive enumeration of all detection results [43]. To achieve faster run times, Ross combined spatial pyramid pooling (SPP-net) and R-CNN, which can share CNN features with each other [44]. Then, Ren proposed a faster R-CNN, which is about 10 times faster than fast R-CNN [45]. Even then, target detection models are still not fast enough for real-time detection. Based on the idea of regression, Redmon proposed You Look Only Once (YOLOv1) [46]. The configuration of YOLOv1 uses GoogLeNet Networks, which effectively perform object detection tasks such as ILSVRC classification and localization. Based on the filter size of 3 × 3, the number of channels is double that in VGG. YOLOv2 is faster and stronger than YOLOv1 [47]. After modification, the Darknet-19 model includes 19 convolution layers and 5 max pooling layers, which can extract objects faster. The configuration of YOLOv3 is based on the Darkney-53 model [48]. YOLOv3′s prior detection system reuses a classifier or locator to perform detection tasks. The model contains many positions and scales; areas made up of position and scale with the highest scores could be considered the test results.

Compared with previous schemes, to eliminate active user actions, this study proposes a new passive visual localization system based on security cameras. This method fuses inter frame and map information at the feature level. During the pedestrian detection period and different from YOLOv3, our improvement is the addition of detection information from the last video frame and the map information to the spatial attention module by area of interest. Experiments show that the performance of this method achieved higher accuracy. However, similarly to scene recognition, target detection only shows us where the pedestrian is at pixel level. Considering that target detection methods only show the pedestrian location at pixel level, we transform the detection results from pixel coordinates to geodetic coordinates by projection transformation (PT). Experiments show that this method can achieve highly accurate positioning results.

## 3. System Overview and Methods

**Figure 1:**
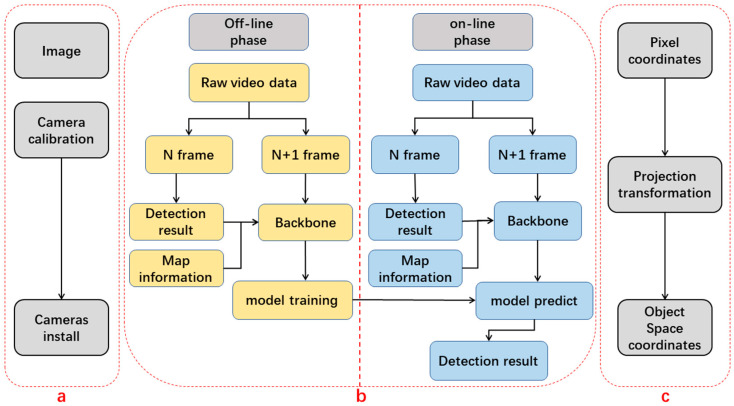
Overview of our passive visual positioning method. The process is composed of (**a**) pretreatment; (**b**) pedestrian detection; (**c**) position estimation.

In this section, we describe the proposed method. The workflow of our visual indoor positioning method is illustrated in Figure 1.

As shown, the proposed system consists of three components: pretreatment, pedestrian detection, and position estimation. These components of the workflow are executed in sequence as follows:

(1) The pretreatment component is shown in Figure 1a. The interior orientation element and lens distortion parameters of a camera are digitized through camera calibration. A security camera is installed with stable localization.
(2) The target detection component is shown in Figure 1b. This includes offline training and online pedestrian detection. Offline training employs a new attention mechanism known as the region of interest attention model (RIAM), which fuses existing detection results and map information.
(3) The position estimation component is shown in Figure 1c. The camera is positioned to look downward so that what is shown in the picture is totally different from the usual pedestrian detection database. After pedestrian detection from the overhead view, the 2D pixel result is used to calculate the location of a pedestrian in plane 2D coordinates, assuming that pedestrians are standing on the ground.

To sum up the proposed system, in pretreatment, camera parameters are calibrated in preparation for further processing. Pedestrian detection includes an off-line and on-line phase. In the off-line phase, the neural network model parameters are tuned using labeled videos, and in the on-line phase, the trained model parameters are used for pedestrian detection. In position estimation, we calculate the user’s position through projection transformation based on the detection results. The following subsections provide the details for each of these components.

### 3.1. Pretreatment

**Figure 2:**
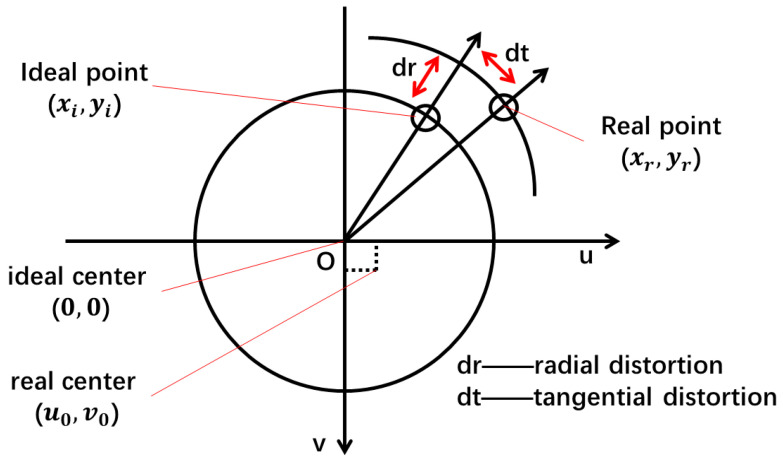
Internal camera distortion problem.

The camera must be calibrated to compensate for internal camera and distortion error. Internal camera error includes focus error and center pixel error in the lens. In addition, the lens distortion error comprises radial distortion and tangential distortion. As shown in Figure 2, the center of focus is offset from the ideal center coordinates. The radial and tangential distortion perpendicular to the radial direction is caused by defects in the shape and fabrication of the lens. Both kinds of distortion error will cause the pixel point to deviate from the ideal position, which will influence localization accuracy.

Therefore, the interior parameters and distortion parameters of the camera are derived to obtain real pixel coordinates. The relationship between the pixel coordinates of the ideal image and those of the actual image is described by Equation (1); here, two tangential and three radial distortions are considered:

(1) $$\{\begin{array}{c} x_{d}=x_{u}\left(1+k_{1}r^{2}+k_{2}r^{4}+k_{3}r^{6}\right)+2p_{1}x_{u}y_{u}+p_{2}\left(r^{2}+2x_{u}^{2}\right)\\ y_{d}=y_{u}\left(1+k_{1}r^{2}+k_{2}r^{4}+k_{3}r^{6}\right)+p_{1}\left(r^{2}+2y_{u}^{2}\right)+2p_{2}x_{u}y_{u} \end{array}$$

where $\left(x_{u},y_{u}\right)$ is the original pixel coordinate, $\left(x_{d},y_{d}\right)$ is the corrected pixel coordinate, $\left\{k_{1},k_{2},k_{3}\right\}$ is the parameter of the radial distortions, $\left\{p_{1},p_{2}\right\}$ is the parameter of the tangential distortions, and *r* is the radius of the pixel.

**Figure 3:**
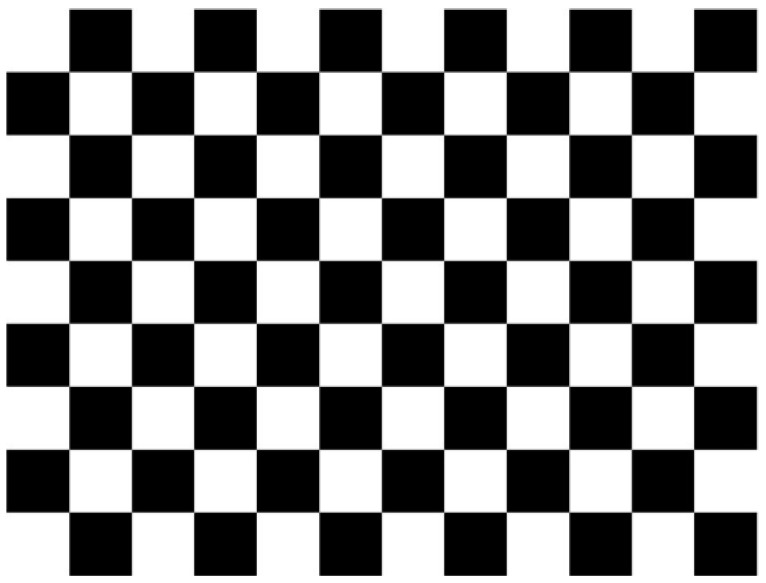
Camera calibration based on chessboard structural geometry.

**Figure 4:**
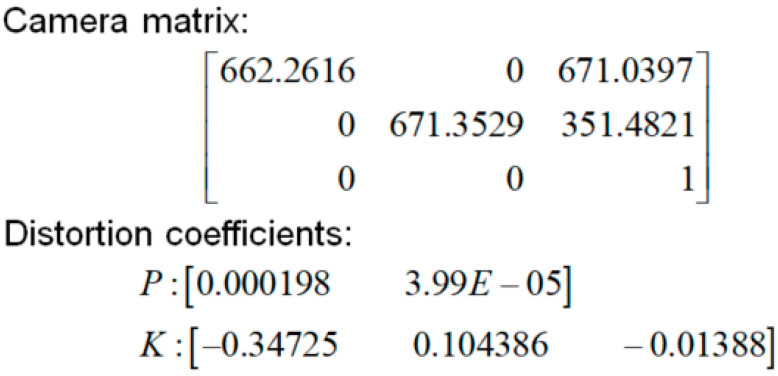
Internal camera parameter matrix.

The calibration method proposed by Zhang [49] was adopted as it delivers high calibration accuracy and is robust, with concise calibration operation and low hardware requirements. This approach uses the structural geometry of a chessboard (Figure 3) to calibrate internal camera parameters (Figure 4). As illustrated in Figure 4, the image center point of the security camera was located at the image coordinate (671,351), while the average focal length f was 666.81. In this study, the calibration error was 0.23 pixels.

### 3.2. Target Detection

Target detection relies on a basic neural network (YOLOv3) that is robust and can tackle different target sizes. During indoor positioning, we have the previously detected result and entrance and exit information of a CAD map of a target building. YOLOv3, however, does not use all the available information and cannot meet the accuracy requirements for passive visual positioning. Thus, a new region of interest attention module (RIAM) was designed and added to YOLOv3 to make full use of the previously detected result and entrance and exit information of a CAD map of a target building. A YOLOv3 deep convolutional neural network, extended by RIAM, is the foundation for our pretrained model to detect pedestrian pixel positions from videos.

#### 3.2.1. Deep Convolutional Neural Networks

**Figure 5:**
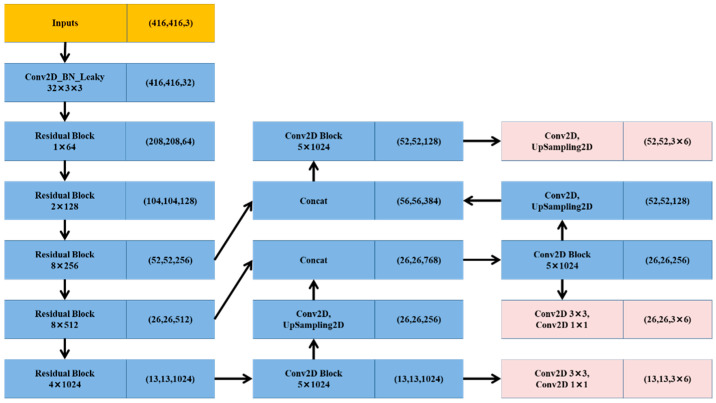
Structure of YOLOv3.

Methods such as YOLO, which use a regression model and entire images as the input of the network, directly return a target border and classify and extract targets in locations in the image. However, YOLOv1 still has many problems and cannot extract small targets. Therefore, we use YOLOv3, as shown in Figure 5.

Unlike other CNN models, a single neural model in YOLOv3 was used for the whole process. This model divides images into different areas, and predicts bounding boxes and probabilities for targets weighted by the probability of the prediction. In contrast to classifier-based systems, YOLOv3 looks at the entire image during testing, so its predictions exploit the global information in the image. Unlike R-CNN, which requires thousands of images of a single target, YOLOv3 makes predictions through a single network assessment. Unlike Faster-RCNN, SSD, YOLOv1, and YOLOv2, this network has three scale factors. Thus, we chose YOLOv3 as the basic network and added the new attention module to improve detection accuracy.

#### 3.2.2. Region of Interest Attention Module

The new region of interest attention module (RIAM) uses the channel attention model as usual; however, the spatial attention model considers the positioning environment, fusing the previously detected information and entrance and exit information of a target from a CAD map of a building interior. A channel-first method was adopted to improve YOLOv3′s target detection accuracy, following [50]. Therefore, in this study, we still use the channel first.

**Figure 6:**
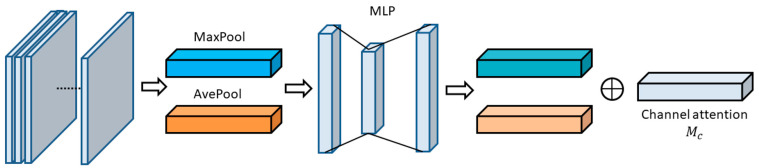
Channel attention.

As shown in Figure 6, the convolutional block channel attention module includes three parts. Firstly, maximum pooling and average pooling are carried out for the input feature graph, and feature compression is carried out along the spatial dimension, so that each two-dimensional feature channel will be compressed into a number. Secondly, the multilayer perceptron (MLP) is used to reconstruct the vectors. Finally, the channel attention weight $M_{c}$ is obtained by the activating function, and RIAM extracts the spatial attention weights from videos.

**Figure 7:**
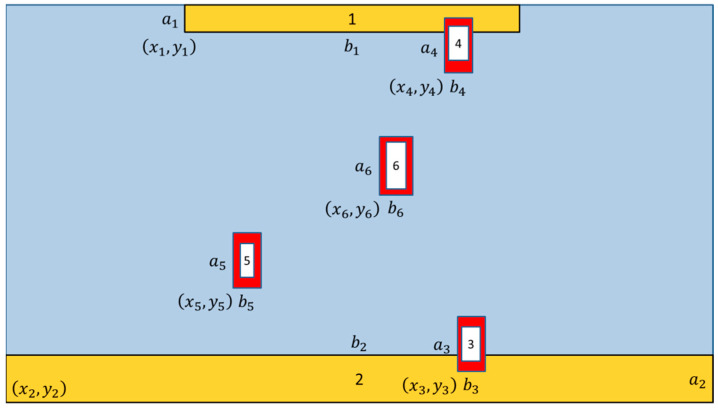
Region of interest.

RIAM combines CAD map information and the previously detected results in our proposed solution for passive visual positioning. Pedestrian positions in one schematic room are shown in Figure 7; the camera is located on the ceiling with six areas of interest, indicating detected pedestrian locations. The two yellow areas are the entrances and exits, and the four white areas are the pedestrian detection results extracted from the video images collected by the camera. The four red areas are the areas based on predictions from the white areas. The pixel size for each area needs four parameters $\left\{\left(x_{i},y_{i}\right),\left(a_{i},b_{i}\right)\right\}$, where the coordinate $\left(x_{i},y_{i}\right)$ represents the point in the left and bottom of the bounding box and $\left(a_{i},b_{i}\right)$ represents the length and width of the bounding box for each pedestrian in the room.

The entrances depicted in yellow are where pedestrians enter the room. The four parameters for the two yellow regions of interest $\left\{\left(x_{i},y_{i}\right),\left(a_{i},b_{i}\right),i=1,2\right\}$ were obtained by picking the relevant frame from the video obtained by the camera. These two locations are used to identify the positions of pedestrians as they may enter the room. For the pedestrians already in the room, the white areas indicate actual target pedestrian detection results. The red areas indicate target pedestrian positions predicted from the frame in the video based on the white areas. The size of the red region of interest $\left\{\left(x_{i},y_{i}\right),\left(a_{i},b_{i}\right),i=3,4,5,6\right\}$ is defined as follows:

(2) $$\{\begin{array}{c} a=\frac{{\left(\left(y_{n}+a_{n}\right)-\left(y_{n-1}+a_{n-1}\right)\right)}^{2}}{y_{n}-y_{n-1}}+a_{n}+y_{n}-y_{n-1}\\ b=\frac{{\left(\left(x_{n}+b_{n}\right)-\left(x_{n-1}+b_{n-1}\right)\right)}^{2}}{x_{n}-x_{n-1}}+b_{n}+x_{n}-x_{n-1} \end{array}\;$$

**Figure 8:**
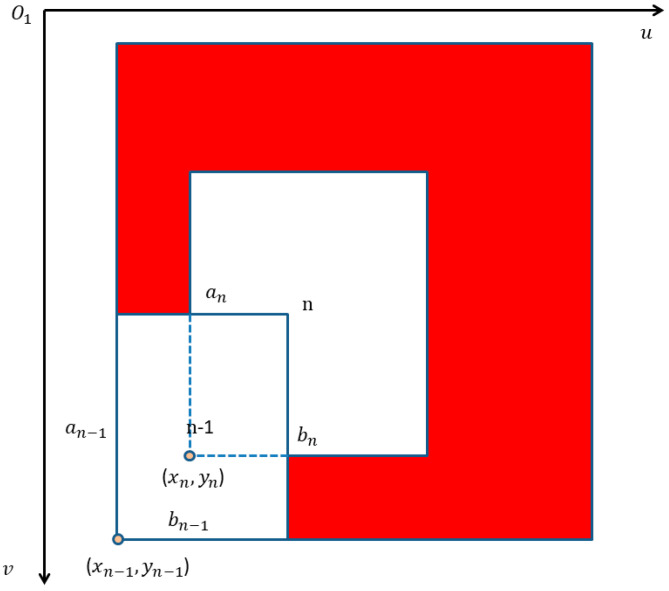
Red region of interest.

As shown in Figure 8, $\left(x_{n-1},y_{n-1}\right),\;\left(x_{n},y_{n}\right)$ are the *n* − 1 and *n* frame’s detection results. Due to the pedestrians walking in a continuous manner, $\left(x_{n+1},y_{n+1}\right)$ is equal to $\;\left(x_{n-1},y_{n-1}\right)$.

Based on the above equations, we can obtain the RIAM. Since the backblock of YOLOv3 is responsible for image feature extraction [48], the RIAM is added between each convolution of backblock to test performance.

### 3.3. Position Estimation

The detection results only indicate who is in the area; however, in a large-scale scenario, we need to know the exact position of each pedestrian. We calculate the coordinates of each pedestrian in the object space coordinate system from pixel coordinates of the pedestrian detection results by projection transformation (PT). However, there is a missing scale problem when calculating the 3D position of a target from a single image. In this application scenario, we assume that pedestrians’ feet are always on the ground. We use the height (H) of the camera to solve the scale problem.

**Figure 9:**
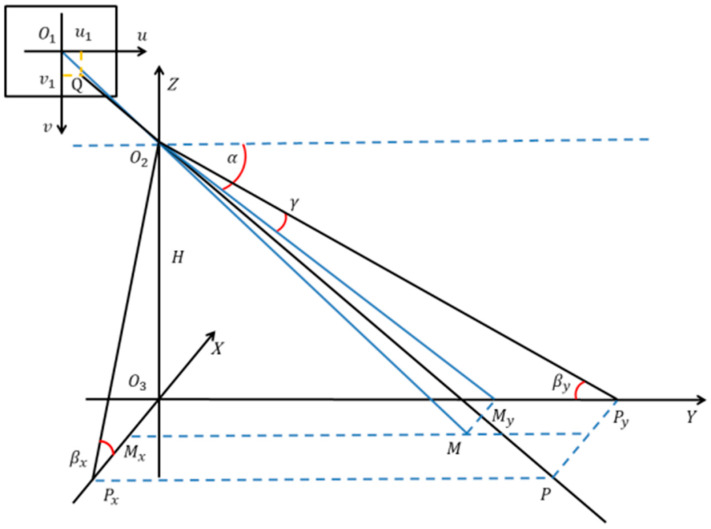
Projection transformation for estimating a target position.

Figure 9 illustrates the corresponding relations between image plane coordinates and object space coordinates. The image plane coordinates have $Q_{1}$ as the origin and the object space coordinate system has an origin at $Q_{3}$. Point $Q_{2}$ is the center point from a pinhole image from the camera, point $O_{1}$ is the projection of point $\left(M_{x},M_{y}\right)$ onto the image plane, and point Q is the projection of point $\;P\left(P_{x},P_{y}\right)$ onto the image plane. According to the projection relationship, we can calculate the square coordinate of pedestrians based on the pixel coordinates detected for pedestrians.

The value {α,γ,β} is used to estimate pedestrian positions in object space coordinates and can be calculated as follows:

(3) $$\{\begin{array}{c} \alpha =∠O_{2}M_{y}O_{3}=\tan^{-1}\left(\frac{H}{O_{3}M_{y}}\right)\\ \gamma =∠M_{y}O_{2}P_{y}=\tan^{-1}\left(\frac{O_{1}v_{1}\times ypix}{f}\right) \end{array}$$

where *H* is the height of the security camera, $O_{3}M_{y}$ is the value of m on the y coordinate, $O_{1}v_{1}$ is the value of Q on the v coordinate, ypix is the actual length of a single pixel module, and f is the focal length of the camera.

We can then obtain the  β:

(4) β=α−γ

We obtain the coordinates of pixel point Q in the object space coordinate system as follows:

(5) $$\{\begin{array}{c} X=O_{3}P_{x}=\frac{H}{\tan\beta_{x}}\\ Y=O_{3}P_{y}=\frac{H}{\tan\beta_{y}} \end{array}$$

where $\beta_{x}$ and $\beta_{y}$ are the angles in the *X*-axis direction and *Y*-axis direction, respectively.

In summary, in pedestrian detection, we sufficiently utilize the previous detection results and CAD map information by coupling them by RIAM. Since we need to provide location services, the target detection result alone is not enough for indoor positioning. We estimate the position of detected pedestrians based on projection transformation. To test the availability of passive visual positioning, we conducted two experiments, as set out in Section 4.

## 4. Experiments

### 4.1. Experimental Environment and Setup

**Figure 10:**
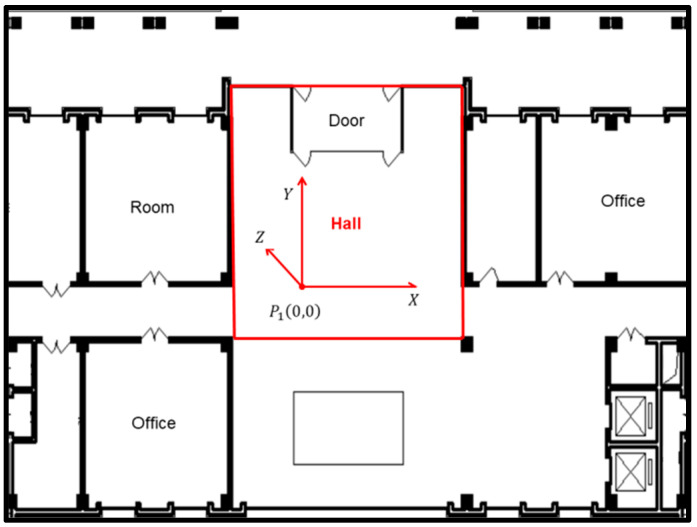
Experimental environment.

The experimental environment was a typical hall in the Sirindhorn Research Center at Wuhan University. Figure 10 is the floor plan of the entire test site; we chose the hall for experiments, marked in red. As shown in Figure 10, we chose point $P_{1}$ as the origin of the object square coordinate system. The *X*-axis goes from left to right and the *Y*-axis and *X*-axis are on the horizontal ground, where the *Y*-axis goes from $P_{1}$ to the door and the *Z*-axis goes from bottom to top.

**Figure 11:**
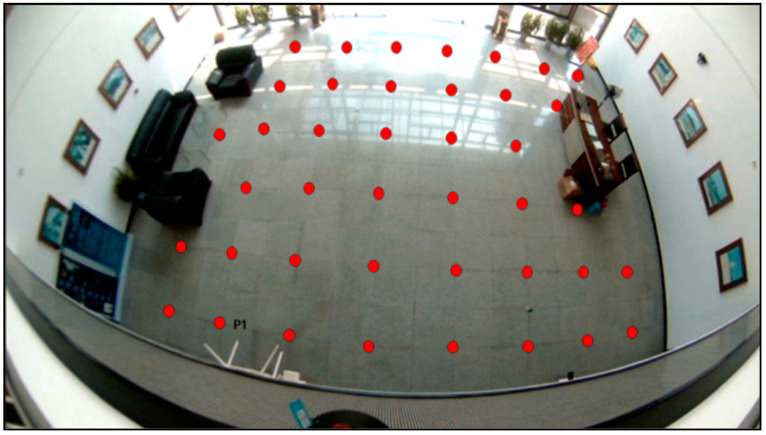
Test-points and camera deployment.

The camera is a top view, so the height will affect visual range. The height of the room or the office is about 3 m, so we chose the hall as the experiment site. The indoor height of the hall is about 10 m, and about 12 m in length and 8 m in width. Moreover, we installed the camera at an altitude of about 7 m in a top-down pose so that the whole hall can be photographed. Furthermore, Figure 11 shows the layout of the experiment site. Due to the sofa and table in the hall, the 41 test-points shown in red dots were spaced regularly about 120 cm apart and were used to evaluate the positioning accuracy of passive visual positioning.

We tested the positioning accuracy for each test-point. The whole experiment ran on a computer with an Ubuntu 16.04 system. The CPU was an Intel Core i7-6700k, the RAM was 32 GB, and the graphics card type of this computer was an NVidia GTX 1070 with 8 GB of memory.

### 4.2. Pedestrian Detection Results

In this study, because the image had a top view, the training set also had to have a top view. However, there are few public top view pedestrian data sets for training. Thus, we built a top view video data set of pedestrians, containing 2000 frames in total, which includes 500 crowd-source frames from videos and 1500 frames collected in three areas.

**Figure 12:**
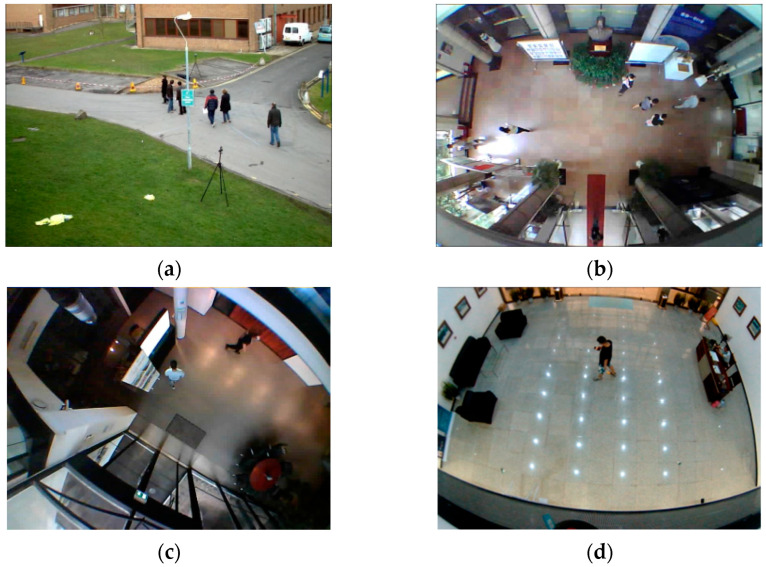
Database: (**a**) MOT15; (**b**) laboratory at Wuhan University; (**c**) hall of the Finland Geodetic Survey Institute; (**d**) hall of the Sirindhorn Research Center.

As shown in Figure 12, there are various pedestrians in various scenarios. Figure 12a is the crowd-source picture, and Figure 12b–d were taken by us. The video as shown in Figure 12a is part of the MOT15 database, and there are about eight pedestrians in the video. The video as shown in Figure 12b was taken in the hall of the laboratory at Wuhan University, and there are seven pedestrians in the video. The video as shown in Figure 12c was taken in the hall of the Finland Geodetic Survey Institute, and there are two pedestrians in the video. The video as shown in Figure 12d was taken in the hall of the Sirindhorn Research Center, and there are two pedestrians in the video.

To test our proposed detection method, Faster-RCNN, SSD512, YOLOv3, and YOLOv3 + RIAM were used for pedestrian detection from the top view. A total of 1400 frames in continuous time were selected as the training set, and the remaining 600 frames in continuous time were used as the test set. The detection accuracy measured by mean average precision (mAP) and the running time for each tested model are shown in Table 1.

As indicated in Table 1, the SSD model had the lowest detection accuracy, with a mAP score of 75.59%, and Faster-RCNN achieved a mAP score of 78.33%. Due to the three preset boxes in different scales, YOLOv3 outperformed the SSD model, with 81.63% detection accuracy. Due to the attention mechanism of the region of interest attention module, YOLOv3 + RIAM delivered the highest performance, achieving 83.49% accuracy. This method yielded a 1.56% improvement over YOLOv3. Although the YOLOv3 + RIAM model used more time than YOLOv3, the accuracy improvement was more important in indoor positioning. Furthermore, the detection results from the YOLOv3 and YOLOv3 + RIAM models were used in the following positioning experiment.

### 4.3. Indoor Positioning Result

Due to the active visual localization method, which requires users to stop walking and take an image, this method is deeply influenced by image quality and may raise privacy issues. In this study, we propose that the passive visual localization method can not only overcome these limitations, but can also achieve the requirement for high accuracy indoor positioning. An experiment was designed to evaluate the performance of the passive visual localization method. The performance of passive visual technology was evaluated using Equation (6) as follows:

(6) $$E=\|P_{true}-P_{loc}\|$$

where $P_{true}$ and $P_{loc}$ represent the two-dimensional true coordinates and the positioning coordinates to be evaluated, respectively; E represents positioning error.

In our experiment, we employed the above detection technology in videos for about one and a half minutes at each test-point. We obtained a frame every second from the videos, so there are 90 frames for each test-point. Then, we calculated the 90 pedestrian locations at each test-point, and we estimated the error of passive indoor positioning by the truth coordinate of the test-points.

**Figure 13:**
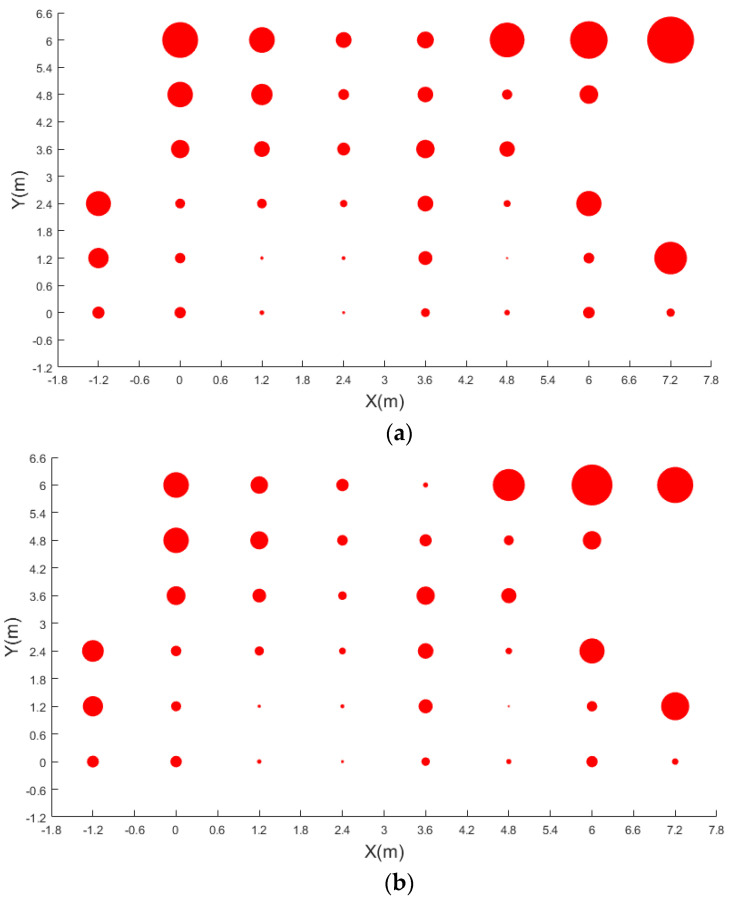
Position error of the 41 test-points. (**a**) Results by YOLOv3; (**b**) results by YOLOv3 + RIAM.

Figure 13 is the position result achieved by detection result and projection transformation (PT). The results of Figure 13a were calculated by YOLOv3, and the results of Figure 13b were calculated by YOLOv3 + RIAM. As shown, there are 41 positioning results of the test-points, and the size of each red point corresponds to the positioning error of each position. It can be seen that the positioning accuracy of the control points close to the projection center is higher than that of the edge. Comparing the two images, although the positioning mean errors of some test-points in the central region were not significantly different, the positioning mean errors of test-points obtained by the YOLOv3 + RIAM method were lower in the edge region. The max error of YOLOv3 + RIAM is smaller. Detailed positioning results are set out in Table 2; the results include root mean square errors (RMSE), mean errors, max errors, min errors, and range of errors. There are two columns of results below each indicator, and the left line is results of YOLOv3 and the right line is results of YOLOv3 + RIAM.

As seen in Table 2, different points have different positioning accuracy. The minimum mean error of two detection methods are both test-point 26, and the maximum mean error of two detection methods are test-points 31 and 36. Test-point 26 is closest to the projection center and test-points 31 and 36 are far from the projection center. Among the 41 test-points of the passive visual positioning based on YOLOv3, there were 27 points with an average accuracy smaller than 0.5 m, while 37 test-points were smaller than 1 m. Moreover, there were 28 points with an average accuracy smaller than 0.5 m, while 39 test-points were smaller than 1 m based on YOLOv3 + RIAM. The results show that YOLOv3 + RIAM can achieve higher positioning accuracy in passive visual indoor positioning. Additionally, the positioning accuracy of passive vision can reach 1 m in 95% of the coverage area.

**Figure 14:**
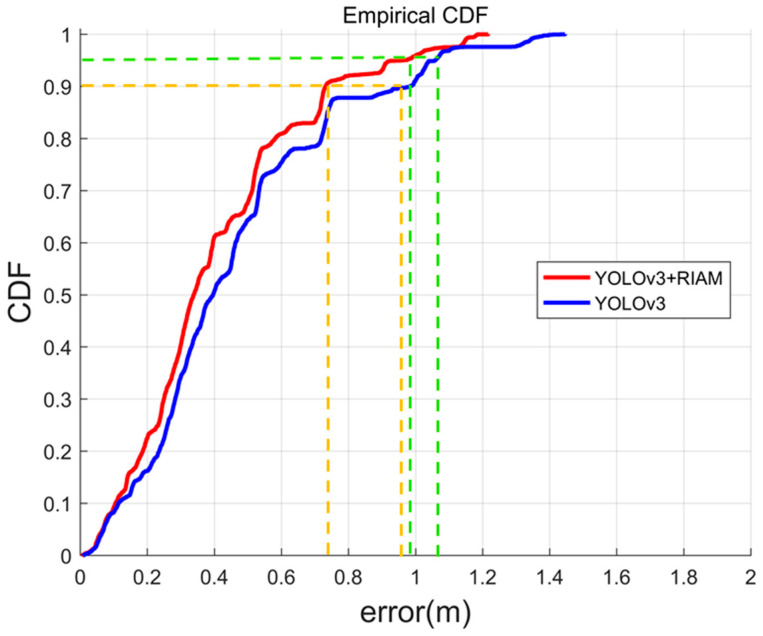
CDF of the whole positioning error.

Figure 14 and Table 3 show all the statistical results and the cumulative distribution function (CDF) of the positioning error. The red curve represents the passive visual localization results based on YOLOv3 + RIAM, and the blue curve represents the results based on YOLOv3. The yellow curve represents the 90% errors and the green curve represents the 95% errors of two methods. YOLOv3 + RIAM performed better in indoor positioning than YOLOv3, especially in the edge region, and the positioning errors of 90% and 95% of RIAM methods were smaller than those of YOLOv3. The RMS error of the positioning method is 1.217 m and 1.446 m and the maximum error of the positioning method is 0.480 m and 0.549 m. The passive vision positioning methods based on YOLOv3 + RIAM achieve an RMSE, 95% error, 90% error, ME, Max, and Min error of 0.480 m, 0.964 m, 0.732 m, 0.404 m, 1.217 m, and 0.006 m, respectively. It is obvious that the proposed detection method performs well in indoor positioning and that passive visual positioning can achieve high positioning accuracy.

Then, we performed a dynamic test in the hall of the Sirindhorn Research Center in order to compare the performance of the proposed method, pedestrian dead reckoning (PDR), and magnetic field matching (MM).

**Figure 15:**
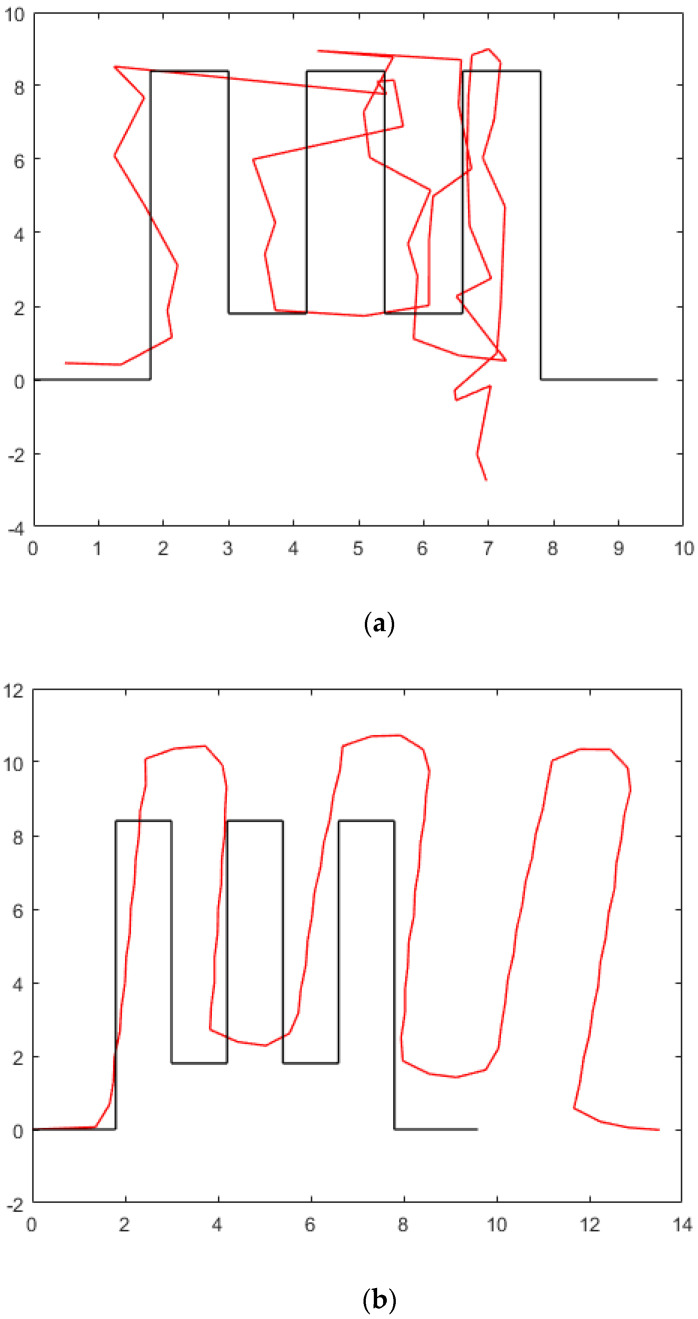

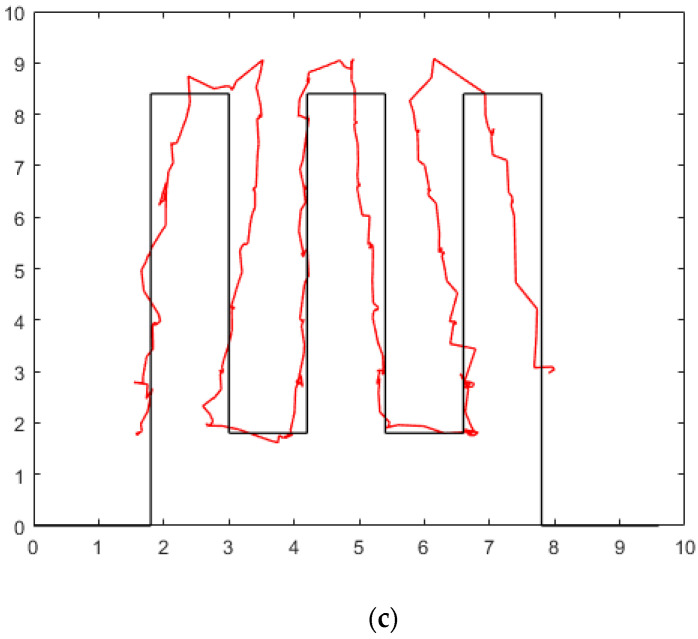
Test-points and camera deployment. (**a**) MM. (**b**) PDR. (**c**) Passive visual.

As shown in Figure 15, the black line is the pedestrian real route, and the red line is the result achieved by the positioning method. Figure 15a is the result achieved by MM, and MM caused a very large positioning error due to mismatching in the hall [51]. Figure 15b is the result achieved by PDR, and we can see that PDR is affected by the position drift error of the inertial navigation system; pure PDR needs other methods to reduce the drift error [52]. Figure 15c is the result achieved by passive visual positioning. Due to the size of the area covered by the camera, the red part is the part with results, and the blue part is the part without results. However, we can see that our method can achieve better positioning accuracy in the hall.

**Figure 16:**
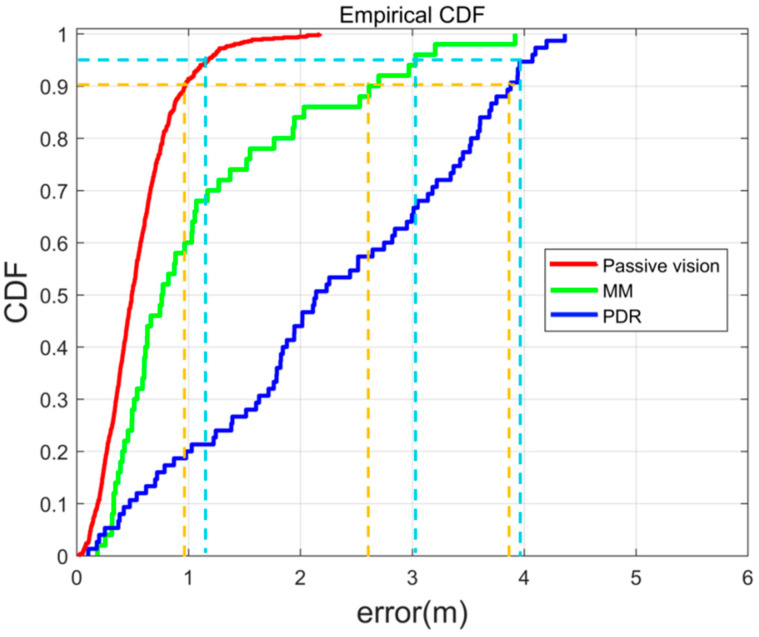
CDF of various positioning methods.

Figure 16 and Table 4 show all the statistical results and the CDF of the positioning error. The red curve represents the passive visual localization results based on passive vision, the green curve represents the results based on MM, and the blue curve represents the results based on PDR. The orange and light blue lines represent the 90% and 95% errors, respectively. The passive visual method performed better in indoor positioning than PDR and MM. The positioning errors of 90% and 95% of the passive visual method were smaller than those of PDR and MM. The RMS error of the positioning method is 0.643 m, 2.581 m, and 1.4351 m. The maximum error of the positioning method is 2.175 m, 4.364 m, and 3.919 m. It is shown that our methods can perform well in the hall environment. Furthermore, in contrast to the active vision positioning method, our approach does not require the user to take a photo, nor does it require a database of images for every place.

## 5. Conclusions

Active visual users must take a photo with their smartphone to obtain location information, raising confidentiality and privacy issues. To solve this problem, we propose passive visual positioning technology based on pedestrian detection and projection transformation. However, the pedestrian in a video frame is collected by a top view camera, which causes the pedestrian to have little textural information, and the detection result cannot achieve accuracy requirements for indoor positioning. To solve this problem, we collected the top view video and improved YOLOv3 with a new attention model containing frame detection results and CAD map information. The detection experiment results show that the proposed method achieves high detection accuracy with a mAP score of 83.49%, which is 1.56% higher than YOLOv3. Compared to active visual positioning technologies, this method does not need users to take photos or a server to build an image database. The proposed method also delivered more accurate indoor positioning results than PDR or magnetic field matching. These results demonstrate that passive visual positioning technology is effective. The CDF error of static positioning is within 0.96 m at 95% and the RMS error of positioning is 0.48 m. The CDF error of dynamic positioning is within 1.168 m at 95% and the RMS error of positioning is 0.643 m. When the number of pedestrians increases, since pedestrian detection based on YOLOv3 + RIAM is calculated based on a regression model, it will not increase the volume of pedestrian detection calculations. In addition, the positioning calculation for each person is calculated separately. The running time to calculate the position of 100 people is 0.00274 s. This result indicates that an increase of the number of people does not have a large impact on the computational complexity. In the future, we will work on the following problems. When there are many pedestrians, if different pedestrians are identified, location information can be sent to the corresponding pedestrians. At present, we match the status and location information of pedestrians on the server with the information on the mobile phone of pedestrians. Furthermore, the MAC address of the pedestrian’s mobile phone can be obtained. Through this matching process, we can use the MAC address to identify unique users, and we can send each user’s location to their mobile phone using the Mac address. If pedestrians block each other, then we can add the training data of the same situation to give the deep learning network more advanced detection ability. In addition, multiple indoor security cameras can be used to shoot images from multiple angles; thus, we can fuse a few pictures to reduce the influence of occlusion on positioning calculations. Furthermore, considering the coverage of the security cameras, we will focus on the extensibility of passive visual localization to provide more universal applications and even higher positioning accuracy.

## Figures and Tables

**Table 1:** Various neural network results.

Model	mAP	Running Time
Faster-RCNN	78.33%	196.2 ms
SSD	75.59%	64.9 ms
YOLOv3	81.63%	29.3 ms
YOLOv3 + RIAM	83.49%	38.3 ms

**Table 2:** Various results for the evaluated 41 test-points.

Num	YOLOv3|YOLOv3 + RIAM Errors (m)									
	RMS		Mean		Max		Min		Range	
Test-Point 1	0.32	0.33	0.32	0.33	0.34	0.35	0.31	0.31	0.04	0.04
Test-Point 2	0.29	0.30	0.29	0.30	0.36	0.38	0.25	0.26	0.11	0.12
Test-Point 3	0.30	0.28	0.30	0.28	0.31	0.30	0.29	0.27	0.03	0.03
Test-Point 4	0.53	0.53	0.53	0.53	0.55	0.55	0.52	0.52	0.02	0.03
Test-Point 5	0.72	0.73	0.72	0.73	0.74	0.76	0.70	0.71	0.04	0.04
Test-Point 6	0.72	1.03	0.72	1.03	0.74	1.04	0.72	1.02	0.02	0.02
Test-Point 7	0.49	0.74	0.49	0.74	0.52	0.76	0.46	0.71	0.06	0.06
Test-Point 8	0.51	0.62	0.51	0.62	0.53	0.64	0.49	0.59	0.05	0.05
Test-Point 9	0.39	0.45	0.39	0.45	0.40	0.46	0.38	0.44	0.02	0.02
Test-Point 10	0.26	0.28	0.26	0.28	0.29	0.30	0.24	0.26	0.05	0.05
Test-Point 11	0.08	0.08	0.08	0.08	0.11	0.11	0.04	0.05	0.07	0.07
Test-Point 12	0.12	0.12	0.11	0.12	0.16	0.17	0.09	0.10	0.07	0.07
Test-Point 13	0.06	0.07	0.06	0.07	0.10	0.11	0.03	0.04	0.07	0.07
Test-Point 14	0.11	0.11	0.10	0.10	0.20	0.19	0.04	0.03	0.17	0.16
Test-Point 15	0.20	0.21	0.20	0.21	0.21	0.23	0.18	0.20	0.03	0.03
Test-Point 16	0.25	0.37	0.25	0.37	0.27	0.40	0.22	0.35	0.05	0.05
Test-Point 17	0.30	0.32	0.30	0.32	0.38	0.40	0.24	0.25	0.15	0.15
Test-Point 18	0.35	0.46	0.35	0.46	0.40	0.51	0.29	0.40	0.11	0.11
Test-Point 19	0.13	0.49	0.13	0.49	0.20	0.55	0.09	0.44	0.11	0.11
Test-Point 20	0.35	0.45	0.35	0.45	0.42	0.52	0.26	0.36	0.16	0.17
Test-Point 21	0.52	0.53	0.52	0.53	0.53	0.54	0.48	0.49	0.04	0.05
Test-Point 22	0.44	0.45	0.44	0.45	0.46	0.48	0.43	0.44	0.04	0.04
Test-Point 23	0.40	0.40	0.40	0.40	0.46	0.46	0.38	0.38	0.08	0.08
Test-Point 24	0.24	0.26	0.24	0.26	0.25	0.27	0.23	0.25	0.02	0.02
Test-Point 25	0.14	0.15	0.14	0.15	0.15	0.16	0.11	0.13	0.03	0.04
Test-Point 26	0.05	0.05	0.04	0.05	0.08	0.09	0.01	0.01	0.08	0.08
Test-Point 27	0.19	0.20	0.19	0.20	0.25	0.26	0.16	0.17	0.09	0.09
Test-Point 28	0.43	0.45	0.43	0.44	0.53	0.55	0.30	0.32	0.23	0.23
Test-Point 29	0.28	0.30	0.28	0.30	0.35	0.37	0.22	0.23	0.13	0.13
Test-Point 30	0.90	1.00	0.90	1.00	0.92	1.02	0.87	0.96	0.05	0.05
Test-Point 31	1.15	1.08	1.15	1.08	1.22	1.14	1.13	1.05	0.09	0.09
Test-Point 32	0.52	0.54	0.52	0.54	0.54	0.56	0.51	0.52	0.04	0.04
Test-Point 33	0.71	0.73	0.71	0.73	0.73	0.75	0.69	0.71	0.04	0.04
Test-Point 34	0.30	0.31	0.29	0.31	0.34	0.36	0.26	0.28	0.08	0.08
Test-Point 35	0.32	0.34	0.32	0.34	0.36	0.38	0.29	0.30	0.07	0.07
Test-Point 36	1.02	1.34	1.02	1.34	1.12	1.45	0.97	1.29	0.15	0.15
Test-Point 37	0.18	0.24	0.18	0.24	0.20	0.26	0.17	0.22	0.03	0.04
Test-Point 38	0.80	0.94	0.79	0.94	0.99	1.14	0.72	0.86	0.27	0.28
Test-Point 39	0.34	0.35	0.34	0.35	0.39	0.41	0.23	0.25	0.16	0.16
Test-Point 40	0.57	0.59	0.57	0.59	0.63	0.65	0.52	0.53	0.11	0.11
Test-Point 41	0.61	0.72	0.61	0.72	0.66	0.77	0.56	0.67	0.10	0.10

**Table 3:** Various statistical results.

Passive Visual	Positioning Error (m)					
	RMS	95%	90%	Mean	Max	Min
YOLOv3+PT	0.549	1.056	0.952	0.460	1.446	0.013
YOLOv3 + RIAM + PT	0.480	0.964	0.732	0.404	1.217	0.006

**Table 4:** Results of various positioning methods.

Passive Method	Positioning Error (m)			
	RMS	95%	90%	Max
Passive visual	0.643	1.168	0.964	2.175
PDR	2.581	3.960	3.852	4.364
MM	1.435	3.029	2.610	3.919
